# Adaptive and Bounded Investment Returns Promote Cooperation in Spatial Public Goods Games

**DOI:** 10.1371/journal.pone.0036895

**Published:** 2012-05-16

**Authors:** Xiaojie Chen, Yongkui Liu, Yonghui Zhou, Long Wang, Matjaž Perc

**Affiliations:** 1 Evolution and Ecology Program, International Institute for Applied Systems Analysis, Laxenburg, Austria; 2 School of Automation Science and Electrical Engineering, Beihang University, Beijing, China; 3 School of Electronic and Control Engineering, Chang'an University, Xi'an, China; 4 Center for Road Traffic Intelligent Detection and Equipment Engineering, Chang'an University, Xi'an, China; 5 School of Mathematics and Computer Science, Guizhou Normal University, Guiyang, China; 6 State Key Laboratory for Turbulence and Complex Systems, College of Engineering, Peking University, Beijing, China; 7 Department of Physics, Faculty of Natural Sciences and Mathematics, University of Maribor, Slovenia; Universidad Carlos III de Madrid, Spain

## Abstract

The public goods game is one of the most famous models for studying the evolution of cooperation in sizable groups. The multiplication factor in this game can characterize the investment return from the public good, which may be variable depending on the interactive environment in realistic situations. Instead of using the same universal value, here we consider that the multiplication factor in each group is updated based on the differences between the local and global interactive environments in the spatial public goods game, but meanwhile limited to within a certain range. We find that the adaptive and bounded investment returns can significantly promote cooperation. In particular, full cooperation can be achieved for high feedback strength when appropriate limitation is set for the investment return. Also, we show that the fraction of cooperators in the whole population can become larger if the lower and upper limits of the multiplication factor are increased. Furthermore, in comparison to the traditionally spatial public goods game where the multiplication factor in each group is identical and fixed, we find that cooperation can be better promoted if the multiplication factor is constrained to adjust between one and the group size in our model. Our results highlight the importance of the locally adaptive and bounded investment returns for the emergence and dominance of cooperative behavior in structured populations.

## Introduction

The emergence of cooperation among selfish individuals is an intensively studied problem [1], [2]. Traditionally, the problem of cooperation is investigated by means of the game theoretical models of the prisoner's dilemma for pairwise interactions, and more generally public goods game for groups of interacting individuals. In particular, the public goods game is abundant in human society, e.g., protecting the global climate and avoiding overfishing of the oceans [3]–[6]. In the classical public goods game (PGG), individuals engage in multiplayer interactions and decide simultaneously whether to contribute (cooperate) or not (defect) to a common pool. Then the accumulated contributions by cooperators are multiplied by a factor large than one, i.e., the so-called multiplication factor, and finally the resulting assets are shared equally among all group members irrespective of their initial decision. From the perspective of each individual, defection is clearly the rational decision to make as it yields the highest income compared to other members. Thus, selfish individuals should decline to contribute and attempt to free ride on the other players' contributions. However, if nobody decides to invest, the group fails to harvest the benefits of a collective investment, which drives the population into the tragedy of the commons [7]. Actually, the group is most successful if everybody cooperates, and hence the dilemma is caused by the selfishness of individual players.

To study the social dilemma in realistic situations, in the last decade the risk PGG [3], [4], the optional PGG [8]–[12], the threshold PGG [13]–[17], the continuous PGG [18]–[20], and the ecological PGG [21], [22] have been developed based on the classical PGG from the viewpoint of realistic societies. On the other hand, several mechanisms for the evolution of cooperation in the PGG, such as punishment [23]–[33], reward [34]–[38], reputation [34], [37], network reciprocity [39]–[55] have been justified. In particular, complex interaction networks provide a natural and reasonable framework for studying the PGG in structured populations. Within this framework, some aforementioned mechanisms, such as punishment and reward have been further studied [28], [31], [32], [36]. Also, some other factors have been incorporated, such as noise [44], social diversity [39], [41], [46], [47], and success-driven distribution [53]. It is found that social diversity associated with the number and the size of the public goods game as well as the individual contribution to each game can greatly promote the emergence of cooperation [41]. Indeed, social diversity by means of the system's other feature information, e.g., game payoffs [47], [56], teaching activity [39] and preferential selection [46] in strategy updating, have been also demonstrated to facilitate cooperation in the PGG.

It is worth mentioning that the inhomogeneities and social diversity about features of the system are widely existent in human society and animal world, which can characterize the asymmetric and different influence of individuals or interacting environments. However, they are introduced artificially in some previous studies mentioned above. Indeed the inhomogeneities or social diversity can emerge spontaneously via the coevolutionary rules, since the values of property should be not invariable, but evolve based on the state of the system. In the context of evolutionary game theory, the adaptive features are often coupled with the strategy evolution. The coevolution of strategies and features of the model, e.g., individual social ties (for example see Refs. [57]–[65]), noise level [66], [67], payoff matrices [68], [69], capability of strategy transfer [71], [72] and individual learning rules [73], [74], have been investigated in different evolutionary games, especially the prisoner's dilemma game (see [75] for a review). Remarkably, recently Lee et al. further proposed a multiadaptive prisoner's dilemma game where both the payoff matrices and the interaction structure are shaped by the behavior of the agents, and found that such multiadaptive mode can result in the coemergence of hierarchical structure and cooperation [70].

At present, we propose a coevolutionary rule in the PGG. We consider that each interacting group has its own multiplication factor, which evolves based on the local strategy distribution in the group and the global strategy distribution in the whole system. Different from the setting in some previous works [53], [68]–[70], this adaptive multiplication factor is used to measure the local interacting or cooperative environment in each group, rather than individual's feature or the whole system's interaction conditions. Correspondingly, the multiplication factor represents the feedback return of the local investment to the public good, and a larger value of the multiplication factor enables a better investment return [18]. Structured populations provide a competent framework to describe this local feature, which is updated based on the local and the global level of cooperation. In the present study, in order to focus solely on the effects of the adaptive investment returns, we employ a square lattice where the number of group members is fixed and always the same. Due to the diversity of local strategy distribution, the investing cooperators can get together in some groups, whereas in other groups they are sparse. The inhomogeneous distribution can induce different investment returns in different interacting groups, which may correspond to the phenomenon of uneven regional exploitation of the common resources in a society. For example, in some pastures the herdsmen may over-exploit the pasture resource by adding more and more animals to their herd, which may lead to the gradual desertification of the grassland. Correspondingly, the socioeconomic returns from herding in these grazing areas decrease gradually and finally the economic losses are unavoidable. On the contrary, in other pastures the herdsmen may still use the grassland resources while at the same time considering the conservation of the ecosystem, and in such cases a higher socioeconomic return is likely. We adopt the state of the system, i.e., the global cooperation level, as the criterion to measure whether the local cooperative environment is favorable or not. Since the state of the system is evolving simultaneously, here we prefer the dynamical global cooperation level, instead of the static criterion in Refs. [69], [70]. Moreover, in real situations the investment return is variable, but should be somewhat limited by external adjustment. In general, it should be limited in a certain range [76].

In this study, we assume that the enhancement factor in each group is updated based on the differences between the dynamical local and global cooperation levels, and limited between the lower 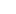 and the upper 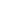 limit. We study how the adaptive and bounded investment returns influence the evolution of cooperation in the spatial PGG. We find that this PGG model with the adaptive and bounded investment returns can effectively enhance cooperation in spatially structured populations, and that appropriately bounded limitations of the multiplication factor can result in the best cooperation level. We find further that, in comparison to the traditionally spatial PGG where only one invariable multiplication factor which is larger than one exists in the whole population, our proposed PGG model can produce a higher cooperation level when each multiplication factor is limited to change only between one and the group size.

## Results

**Figure 1:**
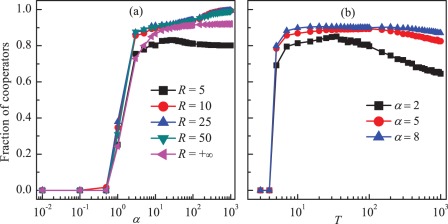
Promotion of cooperation due to adaptive and bounded investment returns. Panel (a) depicts the fraction of cooperators 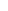 in dependence on the feedback strength 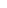 for different values of 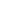. Panel (b) depicts the fraction of cooperators in dependence on the boundary value 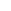 for different values of 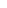. It can be observed that cooperation can be promoted for large values of feedback strength, and there exist moderate boundary values warranting the best promotion of cooperation. Here, 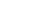.

We start by presenting the results as obtained when the lower limit of the multiplication factor 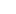 is equal to the opposite number of the upper limit 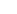, i.e., 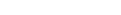. Here 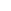 is the limit value (for the detailed definitions see the Methods section). Figure 1(a) shows the cooperation level 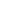 at equilibrium in the population in dependence on the feedback strength 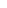 for different values of 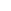. We find that when 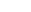 cooperation can be promoted for larger 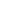. To be specific, when 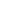 is small, e.g., 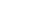, full defection is achieved, irrespective of the values of feedback strength. This is because cooperators cannot survive in structured populations if the maximum enhancement factor is not sufficiently large [44]. When 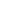 becomes larger, e.g., 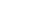, the cooperation level first increases and then slightly decreases. Subsequently, it holds at about 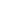 with increasing 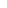. While there is no limitation for the multiplication factor, although the cooperation level is very high, but a small amount of defectors can survive in the population even for high feedback strength, e.g., 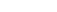. When moderate values of 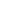 is set, e.g., 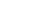, full cooperation can be achieved for high feedback strength, and the cooperation level is very similar for other moderate values of 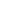. To further qualify the effects of 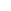 on the evolution of cooperation, we present 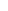 as a function of 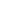 for different 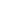 in Fig. 1(b). Clearly, we see that for different values of 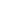, the cooperation level first increases dramatically from zero until reaching the maximum value at a moderate 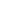, then decreases slowly with increasing 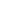. Here, we do not show the cooperation level in dependence on 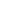 for large 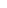. In fact, we can still observe the nonmonotonous dependence of 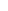 on 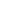 even for large 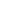. These results suggest that the PGG model with the adaptive and bounded multiplication factor can effectively enhance cooperation in spatial structures, and higher cooperation level can emerges if an appropriate limitation is considered for the dynamical multiplication factor.

**Figure 2:**
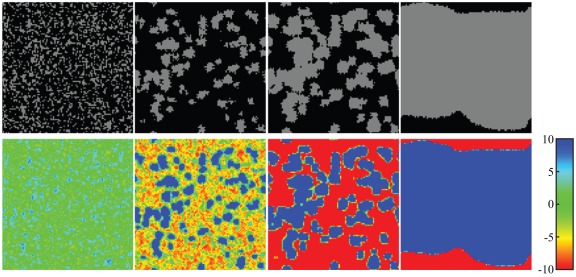
Characteristic snapshots of strategy and multiplication factor distributions on a **square lattice during the coevolutionary process.** Top row depicts the time evolution (from left to right) of typical distributions of cooperators (grey) and defectors (black) on a square lattice, and bottom row depicts the corresponding time evolution (from left to right) of typical distributions of multiplication factor. Results in all panels are obtained for 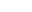, 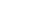, and 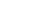. We have checked that similar results can emerge for other parameter settings.

In order to intuitively understand the evolution of cooperation, we show some typical snapshots of the distribution of strategy and multiplication factor in the whole population in Fig. 2. We find that at the beginning of evolution, cooperators can form many small and isolated patches. But subsequently, some small compact cooperator clusters are embedded in the sea of defectors. As time increases, the cooperator clusters increase gradually, and finally cooperators may expand as a single ever growing cluster [upper row in Fig. 2]. Correspondingly, the multiplication factor in the full cooperation group can reach the upper limit, whereas the multiplication factor in the full defection group can reach the lower limit [bottom row in Fig. 2]. However, the multiplication factor within the groups along the boundary of cooperators and defectors reaches a value between the lower and upper limit.

In combination with the above investigations, let us now explain the emergent results. Indeed, a feedback mechanism is at work between the strategy distribution and the distribution of multiplication factors in all the groups. Therefore, cooperators form compact clusters, and these clusters can become larger and larger, especially when the global cooperation level is not very high. Meanwhile, the multiplication factor in the cooperators' clusters becomes larger and larger, which provides cooperators, especially the ones on the boundary, with a higher payoff. Whereas defectors also gather together, and the multiplication factor in the defectors' clusters becomes smaller and smaller [bottom row in Fig. 2]. In those interacting groups where the multiplication factor is negative, it is better for the players to choose the defective strategy such that they can have relatively higher payoffs. In a sense, this adaptive mode can induce a double-edged sword effect on the evolution of cooperation. However, under the social learning defectors are inclined to learn from their neighboring cooperators. As a consequence, the evolution of cooperation can be favored by this locally adaptive investment return.

If there is no limitation for the adaptive multiplication factor, due to the continuing negative feedback effects cooperators cannot invade the defectors' clusters, even if the feedback strength is high. In this situation, cooperators can thrive, but cannot dominate the whole population. When the limitation is considered for the dynamical multiplication factor, the feedback mechanism, especially the negative feedback effects, can be effectively weakened. If there is too much restriction for the adaptive multiplication factor, that is, 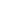 is not very large, e.g., 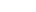, the multiplication factor in some groups can reach the upper limit from a negative value due to the social learning, but this upper limit cannot warrant the promotion of cooperation in spatial PGG even if the feedback strength is enough high. Whereas for a larger 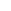, e.g., 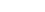, the multiplication factor in the group along the boundary reaching the upper limit can warrant a better promotion of cooperation. Hence, this adaptive and bounded mode for the multiplication factor can provide a better environment for the evolution of cooperation.

**Figure 3:**
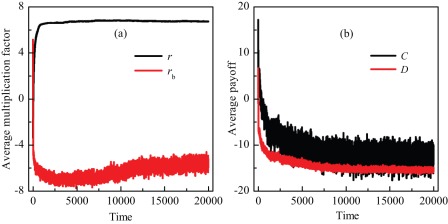
Time evolution of average multiplication factor and payoffs. Panel (a) depicts the time evolution of average values of multiplication factor in the whole population and in the boundary groups, respectively. Panel (b) depicts the time evolution of average payoffs of cooperators and defectors along the boundary, respectively. It can be observed that although the average value of multiplication factor in the whole population is large enough for the evolution of cooperation [44], the average value along the boundary becomes negative. Correspondingly, the average payoffs of cooperators and defectors along the boundary are both less than zero. As time increases, the average payoff of cooperators along the boundary is a little higher than that of defectors, but has larger fluctuations. Here, 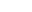, 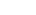, and 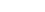.

However, under this adaptive and bounded mode, the multiplication factor in some groups along the boundary cannot rapidly become a positive and large value from a negative one when the feedback strength is not very high [bottom row in Fig. 2]. Although the average multiplication factor 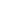 in the whole population can reach an enough high value which can make the cooperation level reach one in the traditionally spatial PGG [44], the average multiplication factor along the boundary of cooperators and defectors 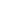 becomes negative as time increases [Fig. 3(a)]. This does not provide a favorable environment for players' interactions. Correspondingly, the average payoffs of cooperators and defectors along the boundary are both negative. Moreover, as time increases the average payoff of cooperators along the boundary is just a litter higher than the one of defectors along the boundary, but have larger fluctuations [Fig. 3(b)]. Under the stochastic strategy updating, defectors do not always successfully imitate their neighboring cooperators, but sometimes may spread their strategy to the cooperators. As a result, cooperators cannot defeat those defectors along the boundary, and they can only coexist with defectors for an exceedingly long time. On the contrary, when the feedback strength is high, by means of social learning the multiplication factor in the group along the boundary can have the opportunity to suddenly reach the upper limit, which can warrant the invading of cooperative behavior into the defectors' clusters. Finally, full cooperation can be achieved.

**Figure 4:**
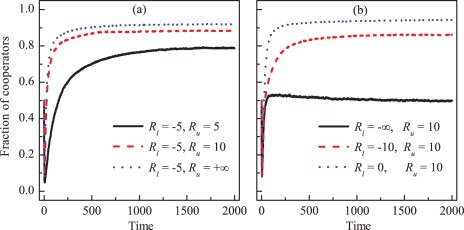
Cooperation promoted when the values of the lower and upper limits of the investment returns are increased. Panel (a) depicts the fraction of cooperators in the whole population as a function of time for fixed lower limit 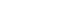 and different values of upper limit. Panel (b) depicts the fraction of cooperators in the whole population as a function of time for fixed upper limit 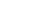 and different values of lower limit. Increasing the values of lower and upper limit can provide more positive effects on the evolution of cooperation. Here, 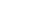 and 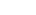.

In what follows, we study how cooperation evolves if the lower limit is not the opposite number of the upper limit. Figure 4(a) shows the typical time evolution of cooperation for fixed 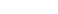 and three different values of 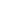. We find that increasing the value of 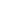 can make the system reach a higher cooperation level, but the cooperation level at equilibrium for 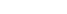 is just slightly larger than the one for 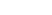. In Fig. 4(b) we show the fraction of cooperators as a function of time for fixed 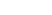 and three different values of 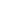. We find that increasing the value of 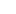 can make the system reach a higher cooperation level. Moreover, as time increases the fraction of cooperators first drops and then rapidly increases, but the larger values of 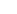 or 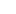 make the cooperation level increases faster. We also find that to have a favorable cooperation level, it is better to set the lower limit higher and it is not necessary to set the upper limit too high.

**Figure 5:**
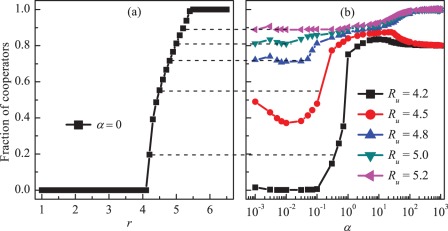
Cooperation promoted even when the investment return is updated within the interval. 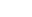
. Panel (a) shows the fraction of cooperators as a function of 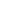 for 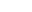. In this situation, the model recovers to the traditionally spatial PGG, where the multiplication factor in each group is fixed at 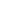 and 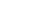. For 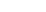, cooperators can survive only if 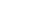, and they can dominate the whole population only if 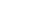. Panel (b) shows the fraction of cooperators as a function of 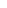 for fixed 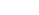 and different values of 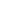. Initially, the multiplication factor in each interacting group is 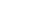. Dash lines are used to indicate the critical value of 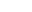 for a better promotion of cooperation in this adaptive and bounded mode for the enhancement factor.

Finally, we study whether cooperation can be better promoted if the adaptive multiplication factor is constrained between 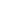 and 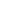 by means of a comparative investigation. Previous work has reported that in the traditional PGG where only one invariable multiplication factor exists in the whole population, defectors outperform cooperators in any given mixed group for 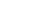 [8]. We further find that in the traditionally spatial PGG, for noise value 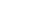 cooperators can dominate the whole population only if 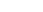, and they can survive in the system only if 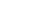, as shown in Fig. 5(a). It is worth pointing out that the traditionally spatial PGG corresponds to the situation of 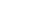 in this present model, where the multiplication factor in each interacting group is fixed at 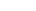. In Fig. 5(b), we set 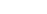, and show the cooperation level as a function of feedback strength 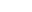 for 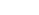 and different values of 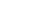. We see that the cooperator density varying with 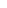 displays two different behaviors: for smaller values of 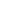, e.g., 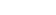, the cooperation level first decreases and then increases until reaching the maximum value. Subsequently, it decreases very slowly with increasing 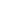 and its value approaches 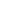; for larger values of 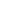, e.g., 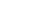, the cooperation level does not change too much for small values of 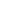, then monotonously increases to one with increasing 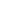. In addition, for smaller values of 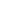 just a small amount of 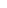 (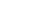) is needed to warrant a better promotion of cooperation in comparison to the traditionally spatial PGG, and the critical amount of 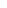 becomes smaller if the 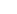 is increased. For larger values of 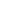, the cooperation level for any value of 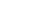 is not less than the one for 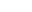. In fact, the average multiplication factor in the population for 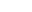 is not larger than the one for 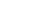, but these results suggest that cooperation can be better promoted in comparison to the traditionally spatial model. In addition, Fig. 5(b) shows that the cooperation level increases with increasing the upper limit of the multiplication factor and the initial value of the multiplication factor. We have also verified that increasing the initial fraction of cooperators is beneficial for the evolution of cooperation in this model.

## Discussion

In summary, we have presented a coevolutionary rule where the multiplication factor in each interacting group is updated based on the local strategy distribution in the group and the global strategy distribution in the whole population, and studied its impact on the evolution of cooperation in the spatial public goods game. We found that this adaptive rule for the multiplication factor can effectively enhance the evolution of cooperation. When the appropriate bounded limitation for the dynamical multiplication factor is further considered, cooperation can be better promoted. In particular, full cooperation can be achieved in the system when the feedback strength is high enough. Also, increasing the lower and upper limit values of the multiplication factor is favorable for the evolution of cooperation, but high cooperation level can be reached even if the upper limit is not very large. We further found that even if the multiplication factor is constrained to change between one and the group size, cooperation can be better promoted in the adaptive mode, in comparison to the classically spatial public goods game where the payoff parameter in each group is fixed and identical.

The adaptive mode for the investment returns results in that a feedback mechanism is at work, that is, the Matthew effect is introduced. From the viewpoint of this emergent feature, our model is related to the one proposed by Perc [53], who considered that the reproductive success of each individual is updated by means of the enforcement of strategy and the distribution of public goods is driven by the reproductive success of individuals. Under this success-driven mechanism, cooperation can be promoted. However, defectors can have a much higher payoff even in the sea of cooperators, and easily enforce their strategy choice to their neighbors. Correspondingly, the superpersistent defector emergences spontaneously, and cooperators cannot dominate the whole population. The complete dominance of cooperators is elusive even if the limitation factor about the value of reproductive success is considered. Whereas in our model, the multiplication factor in each interacting group is updated based on the local and global strategy distribution, which characterizes the local investment environment for collective interactions, rather than individual's personality. Also, we incorporate the limitation factor for the dynamical multiplication factor. In this framework, cooperators and defectors can form their own compact clusters respectively, and correspondingly cooperators along the boundary can have a higher payoff than the neighboring defectors. Under the social learning, cooperators can easily spread their strategy even if the noise level for strategy updating is large. In particular, when the feedback strength is high, the interacting environment including some defectors can rapidly become favorable. Thus, cooperators can gradually invade defector's clusters, and finally dominate the whole population. It could be concluded that our work further enriches the knowledgeless of coevolutionary rules in PGGs, and importantly our spatial PGG model with adaptive and bounded investment returns not only can promote cooperation, but also make cooperators completely dominate the population.

It is worth emphasizing the bounded values of the multiplication factor play a different role in the evolution of cooperation in our model in comparison to the one in Ref. [70]. It is found that the main results remain qualitatively if the limit value of the payoff parameter in the prisoner's dilemma is large enough. Moreover, it is demonstrated that the final cooperation level strongly depends on the values of initial payoff parameter and feedback strength in Ref. [70]. In particular, with increasing the initial payoff parameter, the probability that the system ends in full cooperation state decreases. Whereas in this work, we find that the introduced limitation factor can weaken the Matthew effect, particularly the negative feedback effect on the unfavorable interacting groups, which makes the limit value a crucial model parameter. The limited negative effects can be overcome via social learning. Hence, appropriate limitation can warrant the best promotion of cooperation. In addition, we show that the finial cooperation level not only depends on the the values of initial payoff parameter and feedback strength, but also depends on the limit values. With increasing the initial payoff parameter and the initial fraction of cooperators, cooperation can be better enhanced when the dynamical multiplication factor is limited. In a sense, this work further explores the effects of adaptive and bounded game payoffs on the evolution of cooperation.

During the coevolutionary process, the investment return in most of interacting groups reaches the upper or lower limit due to the feedback effects. This segregation and polarization of investment returns occurs spontaneously over time, which is different from the distribution in Ref. [47]. In the latter case, the distribution of the multiplication factor is artificially introduced by the authors and does not change during the evolutionary process. Although the emergent values of the multiplication factor in all the interacting groups do not display too much diversity, we find that cooperation can be promoted in this adaptive and bounded mode. Compared with the results in the traditionally spatial PGG model, cooperation can be better promoted even if the multiplication factor can only change between one and the group size.

In the present model, we consider the adaptive mode for the multiplication factor in a group based on the local cooperation level in the classical PGG where players just have two discrete strategy choices 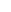 or 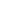, and correspondingly the local cooperation level only has several finite values. To make the local cooperation level change continuously between zero and one, we also introduce the adaptive and bounded investment returns into the spatial continuous PGG [18]–[20], and still find that this PGG with adaptive and bounded investment returns promotes cooperation. We also test our model in well-mixed populations as well as on other types of interactions networks, and still find that cooperation can be enhanced by the proposed coevolutionary rule. Moreover, we would like to point out that in this work we fix the value of noise to one. In general, the qualitative behavior of the system remains unchanged for other values of noise, although for pairwise interactions there may exist an optimal value of noise at which the evolution of cooperation is most successful [44]. It could be inferred that if we can choose the optimal noise value for strategy updating, the positive effects from social learning can be amplified. We also believe that, cooperation can be better promoted if we further incorporate the selection of noise level in strategy adoption [66], [67] into this adaptive and bounded mode for the multiplication factor.

## Methods

We consider the PGG on a square lattice of size 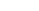 with periodic boundary conditions. Each individual who is a pure strategist can only follow two simple strategies: cooperate (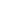) and defect (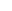). Cooperators contribute a fixed amount (here considered to be equal to 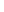 without loss of generality) to the public good while defectors contribute nothing. The sum of all contributions in each group 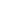 is multiplied by the factor 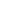, and the resulting public goods are distributed among all the group members. Correspondingly, the payoff of player 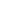 from the group 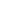 is
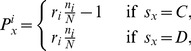
(1)where 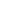 denotes the strategy of player 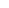, 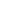 denotes the number of cooperators in the group 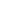, and 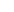 denotes the group size. Here, we consider the square lattice with von Neumann Neighborhood. Accordingly, the interacting group size is fixed at 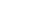, and each individual belongs to five different groups. The payoff of each player is accumulated from the fixed five interacting groups, and thus player 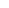
' total payoff 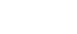.

After playing the games, the multiplication factor in each group needs to be updated. Specifically, we assume that the multiplication factor of the group centered on player 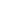 at time 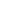 is
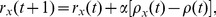
(2)where 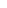 controls the strength of feedback from the comparison between the local and global cooperative environments, 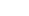 is the multiplication factor of the focal individual 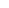
's group at time 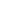, 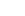 is the fraction of cooperators in the whole population at time 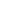, and 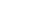 is the local cooperation level in the group centered on 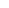 at time 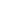. Here, 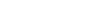, where 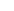 denotes the number of cooperators in the group where player 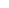 is the focal individual at time 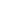. Moreover, we consider the limitation for the adaptive multiplication factor following previous work [70], that is, letting 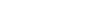 if 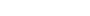 and letting 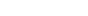 if 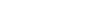. Here, 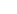 and 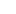 respectively represent the upper and lower limits of the multiplication factor in each group, and they satisfy the following inequalities: 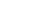 and 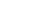. In particular, when 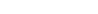, the multiplication factor is constrained in a symmetric interval, which is the same to the setting in Ref. [70]. Moreover, when 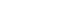 (no bounded limitation for the multiplication factor) or 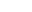 (no updating for the multiplication factor), the average multiplication factor in all the interacting groups 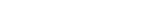, where 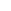 is the initial value of the multiplication factor in each group.

Subsequently, each player is allowed to learn from one of its neighbors and update its strategy. Player 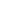 adopts the randomly chosen neighbor 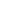
' strategy with a probability depending on the payoff difference as
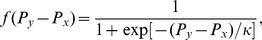
(3)where 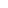 denotes the amplitude of noise [77], accounting for imperfect information and errors in decision making. Following previous work [70], we simply set 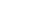 representing that it is very likely that the better performing players will pass their strategy to other players, yet it is possible that players will occasionally learn also from the less successful neighbors.

Simulations of this spatial PGG model are performed by means of a synchronous updating rule, using 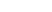 to 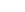 system size. Initially, the two strategies of 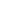 and 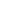 are randomly distributed among the population with an equal probability, and the multiplication factor in each interacting group has the same value 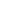. The key quantity for characterizing the cooperative behavior of the system is the density of cooperators, which is defined as the fraction of cooperators in the whole population. The system can reach a dynamical equilibrium after a suitable transient time [78]–[80]. Then the density of cooperators reaches its asymptotic value 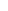 and remains there within small fluctuations (less than 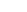). This asymptotic value is taken to describe the cooperation level in the whole population, and all the simulation results are averaged over 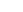 different realizations of initial conditions.
